# Supplementary material for: Bone metabolism factors in predicting the risk of osteoporosis fracture in the elderly
Source: BMC Musculoskelet Disord. 2024 Jun 5;25:442. doi: 10.1186/s12891-024-07560-5 (PMC11155048; doi:10.1186/s12891-024-07560-5)
Supplement: Supplementary file 1 — Supplementary Material 1 [file 12891_2024_7560_MOESM1_ESM.doc]

**Bone metabolism factors in predicting the risk of osteoporosis fracture in the elderly**

Jun Zhang^1#^, Yi Hu^1^, Weifan Cai^1^

1. Department of orthopedic, Changde Hospital, Xiangya School of Medicine, Central South University（The first people’s hospital of Changde city), Changde city 415000,Hunan province, PR. China.

#Corresponding author:

Jun Zhang, NO.818, Renmin Road, Wuling District, Changde city 415000, Hunan province, China. E-mail: [zhangjunchangde@21cn.com](mailto:zhangjunchangde@21cn.com)

**Running title:** Bone metabolism on osteoporosis fracture

**Abstract**

**Objective:** Osteoporosis (OS) is a systemic bone disease characterized by low bone mass and bone microstructure damage. This study aims to investigate the predictive value of combined biochemical indicators of bone metabolism on the risk of OS fracture in the elderly and the association in prognosis.

**Methods:** According to the T value, 88 elderly fracture patients were grouped as the control group (without OS, 43 cases) and observation group (with T value <-2.5, which could be diagnosed as OS, 45 cases). The content of boney containing protein (BGP), total type 1 collagen amino terminal extender peptide (TPINP), β-Crosslaps (β-CTX), parathyroid hormone (PTH) and insulin-like growth factors-1 (IGF-1) was compared. Multivariate logistic regression was adopted to analyze the correlation between biochemical indexes and the occurrence of senile OS fracture and the related risk factors. The diagnostic value in the elderly was analyzed by receiver operating characteristic (ROC) curve.

**Results:** The levels of BGP, TPINP, β-CTX, PTH and IGF-1 were elevated, and the level of IGF-1 was decreased in the observation group compared with the control group (*P*<0.05). The elevated content of BGP, TPINP, β-CTX and PTH, and the decreased expression of IGF-1 were influencing factors for OS fractures in the elderly (*P*<0.05). The sensitivity and specificity to predict the occurrence of OS fractures in the elderly were 91.70% and 90.50%, respectively. The AUC of combined detection was 0.976 (95% CI: 0.952-1.000), which was memorably higher than single indicator detection (*P*<0.05). Among 45 patients, 32 cases had good prognosis and 13 had poor prognosis. In comparison with the good prognosis group, the content of BGP, TPINP, β-CTX and PTH were sensibly higher, the level of IGF-1 was prominently lower, and the proportion of fracture history was much higher in poor prognosis group (*P*<0.05). Fracture history, BGP, TPINP, β-CTX, PTH and IGF-1 were independent risk factors for poor prognosis of elderly OS fractures (*P*<0.05).

**Conclusion:** Bone metabolism factors were associated with poor prognosis of OS in the elderly. The combined detection had higher diagnostic value in calculating the risk of OS fracture in the elderly than single indicator detection.

**Keywords:** Biochemical indicators of bone metabolism; Osteoporotic in the elderly; Fracture; Risk of occurrence; Early prognosis

**1.Introduction**

Osteoporosis (OS) is a systemic bone disease characterized by low bone mass and bone microstructure damage, which mainly occurs in the elderly. In recent years, the incidence rate of OS has also increased with the aging of the population [1]. At present, the treatment plan for patients with OS fracture includes surgical treatment and non-surgical treatment. Among them, the hip fracture with high mortality advocates early internal fixation surgery, which can enable patients to leave bed early and reduce the incidence of postoperative complications and mortality to a certain extent [2]. Because most of the patients are elderly people with other organ diseases, cardiovascular and cerebrovascular accidents are prone to occur during the treatment, which greatly increases the complexity and risk of treatment. Therefore, how to effectively predict the risk of OS fracture in the elderly is of great significance to reduce the occurrence, treatment and prognosis of fracture.

Biochemical indicators of bone metabolism refer to the relevant ions, molecules and regulatory hormones released into the blood or discharged from the urine during bone transformation, which can represent the activity of osteoblasts and osteoclasts, and can also reflect the rate of bone formation or bone absorption [3]. At present, there are many bone metabolic markers to evaluate the risk of OS fracture, such as boney containing protein (BGP), total type 1 collagen amino terminal extender peptide (TPINP), β-Crosslaps (β-CTX), parathyroid hormone (PTH) and insulin-like growth factors-1 (IGF-1), etc. The detection of these above indicators reflects the status of bone turnover, which helps to judge the diagnosis and classification of osteoporosis, and effectively predicts the risk of hip fracture in the elderly. These indicators have been studied in clinical practice, with their advantages and disadvantages and indications, which can provide a theoretical basis for our present study [4]. However, the sensitivity and specificity of the detection of a single biochemical indicator of bone metabolism have certain limitations. At present, there is still no indicator which can accurately predict OS fracture in clinical practice [5].

In this study, 88 cases who met the criteria were finally included. The level of bone metabolism biochemical indicators between non-fracture and fracture was compared to analyze the affecting factors related to the occurrence of OS fracture in the elderly. The predictive value of combined biochemical indicators of bone metabolism on the risk of OS fracture in the elderly and its relationship with poor prognosis were further analyzed.

**2. Materials and methods**

**2.1 Genaral material**

126 elderly fracture patients admitted in our hospital during December 2018 to December 2021 were chosen as the study objects. Inclusion criteria: (1) Those who have not used parathyroid hormone or its analogues or calcitonin, immunosuppressive agents and other drugs that affect bone metabolism recently. (2) Patients with complete clinical data. (3) Patients with normal coagulation function and immune function. (4) Patients ≥ 50 years old. Exclusion criteria: (1) those with abnormal cognitive function and unable to communicate normally. (2) Those with serious primary diseases such as heart, liver and kidney. (3) Patients with other neurological diseases. (4) Those who are allergic to the drugs used in this study. 88 cases who met the criteria were finally included. After being enrolled, the researchers were examined using dual-energy X-ray absorptiometry (DXA) method on the bone mineral density instrument (Hologic Discovery, USA) to measure the bone mineral density (BMD). The measurement results were expressed by T value. T= (BMD value - bone peak value of healthy people of the same gender)/standard deviation of bone peak value of healthy people of the same gender [6]. According to the T value, the selected cases were further grouped as the control group (no OS) with 43 cases and observation group (with T value <-2.5, which could be diagnosed as OS) with 45 cases. Among them, the observation group included 20 males and 25 females, aged 51-82 years, with an average age of (65.73 ± 9.42) years, a body mass index (BMI) of 22-28kg/m^2^, and an average BMI of (24.95 ± 1.66) kg/m^2^. The control group included 19 males and 24 females, aged 50-79 years, with an average age of (63.20±9.13) years, a BMI of 23-28kg/m^2^, and an average BMI of (25.11±1.66) kg/m^2^. There existed no difference in general data such as the age and gender between two groups (*P*>0.05). The selection process of general data was shown in Figure 1. The study was ratified by the Ethics Committee of our hospital, and the patients and their families were informed of the study.

**2.2 Methods**

**Determination of serum biochemical indexes of bone metabolism**

5 ml of venous blood under fasting condition was extracted in the morning, placed in a vacuum centrifuge tube, shaken the tube and fully mixed. The blood was centrifuged at 3000 r/min for 10 min to obtain the upper serum, and the serum was frozen - 20℃ for testing. The content of BGP, TPINP, β-CTX, PTH and IGF-1 was examined using corresponding Enzyme-Linked Immunosorbent Assay (ELISA) kit. BGP kit was from Shanghai Bangjing Industrial Co., Ltd., TPINP β-CTX kit was from Shanghai Ruifan Biotechnology Co., Ltd., PTH kit was from Yixing Biotechnology Co., Ltd., and IGF-1 kit was from Shenzhen Kerunda Biotechnology Co., Ltd. (the detection was carried out in strict accordance with the kit instructions).

**Prognosis assessment**

The patients in the observation group were treated with surgery (hip arthroplasty, etc.). According to the outcome, the patients were further grouped as the good prognosis group (32 cases with no recurrence or no re-fracture) and the poor prognosis (13 cases with death, re-fracture or infection). At the same time, the general data of patients include the age, BMI, fracture history, smoking history, drinking history, BGP, TPINP β-CTX, PTH and IGF-1 levels were collected.

**2.3 Outcome measures**

Comparison of bone metabolism biochemical indicators content: according to the T value, the selected patients were grouped as the control group (without OS) of 43 cases and the observation group (with T value<-2.5, OS can be diagnosed) of 45 cases. The original data of bone metabolism biochemical indicators was collected, and was compared using chi-square test and t-test. The difference was statistically significant with *P*<0.05.

The influencing factors of OS fracture in the elderly: Multivariate logistic regression analysis was used to analyze the influencing factors of OS fracture in the elderly.

The clinical diagnostic value of biochemical indicators of bone metabolism: Receiver operating characteristic (ROC) curve was drawn to analyze the diagnostic value of BGP, TPINP, β-CTX, PTH and IGF-1 alone and in combination for OS fractures in the elderly.

The relationship between biochemical indicators of bone metabolism and prognosis of OS fracture in the elderly: the patients were followed up for 12 months, and were grouped as good prognosis group (with no recurrence or no re-fracture) and poor prognosis group (death, re-fracture and infection) according to the prognosis. The general data of patients (age, gender, BMI, fracture history, smoking history and drinking history) was collected, and the content of bone metabolism indicators was examined. Multivariate logistic regression was adopted to analyze the biochemical indexes of bone metabolism and the risk factors of poor prognosis of senile OS fracture.

**2.4 Statistical analysis**

SPSS21.0 software was used for data statistical analysis. The measurement data was described as ($\bar{x}\pm s$)and compared by t test. The enumeration data was expressed in the form of n (%), and compared following χ ^2^ test. Multivariate logistic regression was employed to analyze the correlation between biochemical indexes of bone metabolism and the occurrence of OS fracture in the elderly and the risk factors for poor prognosis. ROC curve was drawn to analyze the diagnostic value of BGP, TPINP, β-CTX, PTH and IGF-1 alone and in combination for OS fractures in the elderly. *P*<0.05 indicated that the difference was statistically significant.

**3 Results**

**3.1** **Comparison of biochemical indicators of bone metabolism levels**

Compared with the control group, the content of BGP, TPINP, β-CTX, PTH and IGF-1 were markedly higher, and the level of IGF-1 was sharply lower in the observation group (*P*<0.05, Table 1 and figure 2).

**3.2** **Multivariate logistic regression of biochemical indexes of bone metabolism and the occurrence of OS fracture in the elderly**

Multivariate Logistic regression analysis exhibited that the elevated content of BGP, TPINP, β-CTX and PTH, and the decreased expression of IGF-1 were influencing factors for OS fractures in the elderly (*P*<0.05, Table 2).

**3.3** **The predictive value of biochemical indicators of bone metabolism on the occurrence of OS fractures in the elderly**

The ROC curve confirmed that the sensitivity and specificity of combined bone metabolism biochemical indicators to predict the occurrence of OS fractures in the elderly were 91.70% and 90.50%, respectively. The AUC of combined detection was 0.976 (95% CI: 0.952-1.000), which was memorably higher than single indicator detection (*P*<0.05, Table 3 and figure 3).

**3.4** **Prognostic value of bone metabolism biochemical indicators in elderly patients with OS fracture**

Among 45 patients, 32 cases had good prognosis and 13 had poor prognosis. In comparison with the good prognosis group, the content of BGP, TPINP, β-CTX and PTH were sensibly higher, and the level of IGF-1 was prominently lower, and the proportion of fracture history was much higher in poor prognosis group (*P*< 0.05, Table 4).

**3.5** **Analysis of prognostic factors of OS hip fracture in the elderly**

Logistic regression analysis confirmed that fracture history, BGP, TPINP, β-CTX, PTH and IGF-1 were independent risk factors for poor prognosis of elderly OS fractures. Among them, fracture history, BGP, TPINP, β-CTX and PTH were risk factors for poor prognosis of senile OS fracture, and IGF-1 was a protective factor (*P*<0.05, Table 5 and table 6).

1. **Conclusion**

At present, the prevalence of osteoporosis is increasing with the aging of the population, so the differential diagnosis of metabolic bone disease and the early diagnosis of OS are receiving more and more clinical attention [7]. At present, the commonly used methods for clinical detection of OS include histological detection, imaging detection and biochemical technology [8]. These three detection methods all have certain accuracy. Histological detection is innovative, but dynamic detection cannot be performed. Due to the limited precision of imaging detection, OS cannot be detected in time, so biochemical technology has become the preferred inspection method [9].

Biochemical indicators of bone metabolism mainly include calcium and phosphorus metabolism regulation indicators, bone formation markers, bone absorption markers, hormones, etc. Different metabolites indicate the degree of bone formation and bone absorption, which reflects the strength and quality of bone. Therefore, biochemical indicators of bone metabolism are commonly used in the diagnosis of osteoporosis and metabolic bone disease. BGP belongs to gamma-carboxyglutamic-acid-containing proteins and is secreted and synthesized by osteoblasts, which is not easily affected by bone absorption factors. Serum BGP can be used to lean about the activity of osteoblasts, especially the newly formed osteoblasts. The content of BGP varies with the age and bone turnover rate, in a negative correlation. The faster the bone metabolism is updated, the higher the osteocalcin value will be, and vice versa [10]. Some studies believed that the reason why BGP reflected the state of osteogenesis activity was that it could specifically combine with hydroxyapatite under normal physiological conditions, thus forming hydroxyapatite crystals by depositing bone salt to elevate bone salt content and bone strength [11]. Bone tissue is mainly composed of organic matter, inorganic minerals, bone cells and water. There is sufficient clinical evidence to prove that organic matter accounts for about 35% of the backbone weight, of which 90% - 98% comes from TPINP. The amino terminal extended peptide chain of type 1 procollagen peptide is P1NP, and the ratio of P1NP dissociated by specific protease during the conversion of procollagen to collagen is 1:1. Therefore, total P1NP can reflect the activity and osteogenic speed of osteoblasts [12]. The high level of total P1NP indicates abnormal bone metabolism, indicating the risk of OS in patients [13].

β-CTX is an internationally recognized marker of bone resorption in recent years, which is a fragment of collagen released into the blood after degradation in the process of bone reconstruction. Elevated β-CTX content reflects reinforced bone absorption and bone loss, leading to the occurrence of OS and deformable bone disease [14]. PTH is secreted and synthesized by the main parathyroid cells. The main function of PTH is to regulate the metabolism of calcium and phosphorus in the body, thus promoting the increase of blood calcium level and the decrease of blood phosphorus level. The main target organs of PTH are bone and kidney. By mobilizing calcium in bone into the blood, PTH promotes the reabsorption of calcium ions and phosphate excretion of renal tubules, thus increasing the blood calcium concentration and reducing the blood phosphorus concentration [15]. Based on the above view, some studies have suggested that the secretion of PTH is mainly regulated by the concentration of calcium ions in plasma. The secretion of PTH is inhibited when the serum calcium concentration increases, and decreased serum calcium concentration stimulates the secretion of PTH [16]. Excessive secretion of PTH can lead to hyperparathyroidism, increase of blood calcium, secondary osteoporosis, urinary stone and other diseases. It is well known that bone mineral density is the main indicator of osteoporosis, and IGF-1 is the main regulatory factor in the process of bone remodeling. Therefore, some studies suggested that bone mineral density and IGF-1 were closely related [17]. IGF-1 can exert similar effects to insulin metabolism and mitosis by autocrine, endocrine, paracrine and other means, thus stimulating the proliferation and differentiation of chondrocytes. The decrease of IGF-1 level will lead to the reduction of osteoblasts, which will inhibit bone formation and increase the probability of OS [18].

This present study exhibited that the content of BGP, TPINP, β-CTX and PTH were markedly higher and the level of IGF-1 was sharply in the observation group than the control group. Multivariate Logistic regression analysis proved that the elevated content of BGP, TPINP, β-CTX and PTH, and the decreased expression of IGF-1 were risk factors for OS fractures in the elderly. These above results were consistent with the previous foreign and domestic research results, which indirectly indicates that the above bone metabolism indicators can reflect the occurrence of osteoporosis [19-21]. However, the occurrence of OS in the elderly is related to many factors, and there is still no gold standard to predict the risk of OS in the elderly. Therefore, this paper speculated that the predictive value of combined bone metabolism index was higher than that of single index. The present study revealed that the sensitivity and specificity of combined bone metabolism biochemical indicators to predict the occurrence of OS fractures in the elderly were 91.70% and 90.50%, respectively. The AUC of combined detection was 0.976 (95% CI: 0.952-1.000), which was memorably higher than single indicator detection. The above results confirmed the conjecture in this study, and the combined prediction provided certain clinical value for improving the risk of OS fracture in the elderly.

Hip replacement is the main treatment for osteoporosis fracture. Although certain curative effect is achieved, it is easy to have infection, pain, re-fracture and other conditions after operation, which has a negative impact on the prognosis and quality of life of patients. At present, many scholars mainly focus on the treatment effect of hip replacement, but the clinical factors related to the prognosis of osteoporotic fractures have not been unified [20]. Some scholars have proposed that the prognostic nutritional index is an independent risk factor for the elderly patients with OS after hip replacement, which can predict the postoperative risk [22-24]. Based on the above conclusions, the present study speculated that there existed a certain correlation between bone metabolism indicators and the prognosis of OS fractures. The reason is that bone metabolism indicators represent the activity of osteoblasts or osteoclasts, which reflects the rate of bone formation or bone absorption. Therefore, bone condition could be evaluated by detecting bone metabolism indicators. In this study, multivariate logistic regression analysis proved that fracture history, BGP, TPINP, β-CTX, PTH and IGF-1 were independent risk factors which affect the poor prognosis of senile osteoporosis fracture, indicating that there existed a certain relationship between bone metabolism index and the early prognosis of senile OS fracture. However, due to the small sample size and short time for pathological selection, this study still has some limitations. At the same time, due to the lack of reference studies, the results of this study have not been fully applied to clinical practice, and need further discussion and demonstration.

To sum up, the innovation of this paper is to reveal the diagnostic value of single and combined detection of bone metabolism biochemical indicators by analyzing the correlation between bone metabolism biochemical indicators and the occurrence of senile OS fractures and the risk factors of poor prognosis. Due to the limited samples size included in the study and the research time, the most accurate threshold has not yet been proposed, and further research is needed to predict the risk of OS fracture in the elderly. At the same time, previous studies suggested that drinking and smoking could affect the incidence of OS, but this present study failed to reach the results supporting this conclusion, and more related studies need to further demonstrate. However, the combined detection of bone metabolism biochemical indicators improves the diagnostic value of predicting the risk of OS fracture in the elderly, and provides certain accuracy for future research. Biochemical indexes of bone metabolism are closely related to the early prognosis of senile OS fracture. Biochemical indicators of bone metabolism have great potential in predicting fracture, diagnosis and classification, and monitoring curative effect.

**Declarations**

**Funding**

None.

**Data availability statement**

The datasets used and/or analyzed during the current study are available from the corresponding author on reasonable request.

**Competing interests**

The authors declare that they have no competing interests.

**Ethics approval and consent to participate**

All procedures performed in studies involving human participants were in accordance with the ethical standards of the institutional and/or national research committee and with the 1964 Helsinki declaration and its later amendments or comparable ethical standards.

All procedures performed in studies were in accordance with the ethical standards of the ethics committee of Changde Hospital, Xiangya School of Medicine, Central South University(201812).

**Consent for publication**

Informed consent was obtained from all individual participants included in the study. The patients participating in the study all agree to publish the research results.

**Authors' contributions**

Jun Zhang confirmed the authenticity of all the raw data and edited the manuscript, Yi Hu collected data and processed the data. Jun Zhang and Weifan Cai conducted the statistics. Jun Zhang reviewed and revised the article. All authors read and approved the final manuscript.

**Acknowledgements**

Not applicable.

**References**

# Chandran M, Brind'Amour K, Fujiwara S,etal. Prevalence of osteoporosis and incidence of related fractures in developed economies in the Asia Pacific region: a systematic review[J].Osteoporos Int,2023,34(6):1037-1053.

# Sing CW, Lin TC, Bartholomew S, etal. Global Epidemiology of Hip Fractures: Secular Trends in Incidence Rate, Post-Fracture Treatment, and All-Cause Mortality[J].J Bone Miner Res, 2023,38(8):1064-1075.

# Li S, Qiu Y, Teng Z, etal. Research on biochemical indexes of bone metabolism in bipolar disorder: A cross-sectional study with newly diagnosed, drug-naïve patients[J]. J Psychiatr Res, 2022, 151:197-204.

# Ladang A, Beaudart C, Reginster JY, etal. Biochemical Markers of Musculoskeletal Health and Aging to be Assessed in Clinical Trials of Drugs Aiming at the Treatment of Sarcopenia: Consensus Paper from an Expert Group Meeting Organized by the European Society for Clinical and Economic Aspects of Osteoporosis, Osteoarthritis and Musculoskeletal Diseases (ESCEO) and the Centre Académique de Recherche et d'Expérimentation en Santé (CARES SPRL), Under the Auspices of the World Health Organization Collaborating Center for the Epidemiology of Musculoskeletal Conditions and Aging[J].Calcif Tissue Int, 2023, 112(2):197-217.

# Tencerova M, Ferencakova M, Kassem M. Bone marrow adipose tissue: Role in bone remodeling and energy metabolism[J]. Best Pract Res Clin Endocrinol Metab,2021,35(4):101545.

# Tryniszewski W, Kamiński G, Maziarz Z, etal.The assessment of testosterone and radioisotopic index of bone metabolism and bone mineral density in men with testosterone deficiency after one year of testosterone therapy[J]. Nucl Med Rev Cent East Eur,2018,21(1):14-19.

# Khawaja A, Sabbagh P, Prioux J, etal.The Relationships Between Skeletal Muscle Index and Bone Variables in a Group of Young Adults[J]. J Clin Densitom,2021,24(1):78-87.

# Lins Vieira NF, da Silva Nascimento J, do Nascimento CQ, etal. Association between Bone Mineral Density and Nutritional Status, Body Composition and Bone Metabolism in Older Adults[J]. J Nutr Health Aging,2021,25(1):71-76.

# Barron RL, Oster G, Grauer A, etal.Determinants of imminent fracture risk in postmenopausal women with osteoporosis[J].Osteoporos Int,2020,31(11):2103-2111.

# DeLuccia R, Cheung M, Ramadoss R, etal.The Endocrine Role of Bone in Cardiometabolic Health[J].Curr Nutr Rep,2019,8(3):281-294.

# Wang B, Wang H, Li Y, etal.Lipid metabolism within the bone micro-environment is closely associated with bone metabolism in physiological and pathophysiological stages[J].Lipids Health Dis, 2022,21(1):5.

# Xiong L, Chen QQ, Cheng Y, etal. The relationship between coronary artery calcification and bone metabolic markers in maintenance hemodialysis patients[J].BMC Nephrol,2023,24(1):238.

# Messina C, Piodi LP, Rinaudo L, etal.Bone strain index reproducibility and soft tissue thickness influence: a dual x-ray photon absorptiometry phantom study[J].Eur Radiol Exp,2019,3(1):33.

# Zhou J, Liu B, Qin MZ, etal.Fall Prevention and Anti-Osteoporosis in Osteopenia Patients of 80 Years of Age and Older: A Randomized Controlled Study[J].Orthop Surg, 2020,12(3):890-899.

# Ostrowska Z, Ziora K, Oświęcimska J, etal.TGF-β1, bone metabolism, osteoprotegerin, and soluble receptor activator of nuclear factor-kB ligand in girls with anorexia nervosa[J]. Endokrynol Pol, 2016,67(5):493-500.

# Eastman K, Gerlach M, Piec I, etal.Effectiveness of parathyroid hormone (PTH) analogues on fracture healing: a meta-analysis[J].Osteoporos Int,2021,32(8):1531-1546.

# Lim J, Kim YJ, Kim S, etal. Increased Risk of Osteoporotic Fracture in Patients With Autoimmune Hepatitis[J].Am J Gastroenterol,2024,119(1):127-137.

# Tian F, Wang Y, Bikle DD. IGF-1 signaling mediated cell-specific skeletal mechano-transduction[J].J Orthop Res ,2018,36(2):576-583.

# GASCO, V., RONCORONI, L., ZAVATTARO, M., et al. Untreated adult GH deficiency is not associated with the development of metabolic risk factors: a long-term observational study[J]. Journal of endocrinological investigation,2020,43(2):197-207.

# Liang F, Sun L, Wang Y. The Relationship Between GNRI Changes and Bone Metabolism Parameters and the Occurrence of Osteoporosis in Elderly Male Patients with T2D[J].Altern Ther Health Med,2023,29(1):85-89.

# Haffer H, Muellner M, Chiapparelli E, etal. Bone quality in patients with osteoporosis undergoing lumbar fusion surgery: analysis of the MRI-based vertebral bone quality score and the bone microstructure derived from microcomputed tomography[J].Spine J,2022,22(10):1642-1650.

# Long G, Liu C, Liang T, etal. Predictors of osteoporotic fracture in postmenopausal women: a meta-analysis[J].J Orthop Surg Res,2023,18(1):574.

# Trandafir AI, Gheorghe AM, Sima OC, etal. Cross-Disciplinary Approach of Adrenal Tumors: Insights into Primary Aldosteronism-Related Mineral Metabolism Status and Osteoporotic Fracture Risk[J].Int J Mol Sci,2023,24(24):17338.

# Kanis JA, Johansson H, McCloskey EV, etal. Previous fracture and subsequent fracture risk: a meta-analysis to update FRAX[J].Osteoporos Int,2023,(12):2027-2045.

**Tables and figures**

**Table 1 Comparison of biochemical indicators of bone metabolism levels**

**($\overline{X}$±S)**

| Groups | BGP (ng/mL) | TPINP (ng/mL) | β-CTX (μg/mL) | PTH (pg/mL) | IGF-1(μg/L) |
| --- | --- | --- | --- | --- | --- |
| The control group (n=43) | 6.98±1.92 | 44.88±1.85 | 0.17±0.05 | 43.09±14.87 | 804.59±77.19 |
| The observation group (n=45) | 16.68±1.69 | 69.00±7.31 | 0.73±0.11 | 73.26±7.59 | 156.67±21.69 |
| t | 25.185 | 20.998 | 30.502 | 12.066 | 33.796 |
| P | <0.001 | <0.001 | <0.001 | <0.001 | <0.001 |

**Table 2 Multivariate logistic regression of biochemical indexes of bone metabolism and the occurrence of OS fracture in the elderly**

| Factors | Regression coefficient β value | Standard error | Wald χ2 value | P value | OR (95% CI) |
| --- | --- | --- | --- | --- | --- |
| Convention | -15.419 | 3.760 | 16.820 | <0.001 |  |
| BGP | 0.200 | 0.090 | 4.879 | 0.027 | 1.221(1.023～1.458) |
| TPINP | 0.096 | 0.040 | 5.929 | 0.015 | 1.101(1.019～1.190) |
| β-CTX | 4.738 | 1.638 | 8.365 | 0.004 | 114.202(4.605～2832.142) |
| PTH | 0.051 | 0.021 | 5.553 | 0.018 | 1.052(1.009～1.097) |
| IGF-1 | 0.005 | 0.002 | 8.525 | 0.004 | 1.005(1.002～1.008) |

**Table 3 The predictive value of biochemical indicators of bone metabolism on the occurrence of OS fractures in the elderly**

| Factors | AUC | 95% CI | Sensitivity | Specificity | P value |  |
| --- | --- | --- | --- | --- | --- | --- |
| BGP | 0.788 | 0.668-0.889 | 0.756 | 0.703 | 0.011 |  |
| TPINP | 0.728 | 0.613-0.842 | 0.667 | 0.558 | 0.031 |  |
| β-CTX | 0.767 | 0.660-0.873 | 0.689 | 0.627 | 0.018 |  |
| PTH | 0.781 | 0.675-0.887 | 0.793 | 0.725 | 0.012 |  |
| IGF-1 | 0.814 | 0.720-0.908 | 0.824 | 0.784 | 0.006 |  |
| Combined detection | 0.976 | 0.952-1.000 | 0.917 | 0.905 | <0.001 |  |

**Table 4 Prognostic value of bone metabolism biochemical indicators in elderly patients with OS fracture**

| Factors | | Poor prognosis group (n=13) | Good prognosis group (n=32) | χ2/t value | P value |
| --- | --- | --- | --- | --- | --- |
| Age (year) | | 66.92±10.71 | 65.25±8.98 | 0.534 | 0.595 |
| BMI (kg/m2) | | 25.46±1.45 | 24.75±1.72 | 1.309 | 0.197 |
| Gender | Male | 5 | 15 | 0.265 | 0.607 |
|  | Female | 8 | 17 |  |  |
| Fracture history | Yes | 9 | 9 | 0.011 | 0.018 |
|  | No | 4 | 23 |  |  |
| Smoking history | Yes | 7 | 14 | 0.672 | 0.749 |
|  | No | 6 | 18 |  |  |
| Drinking history | Yes | 8 | 16 | 0.528 | 0.356 |
|  | No | 5 | 16 |  |  |
| BGP (ng/mL) | | 24.26±5.30 | 16.51±1.62 | 7.553 | <0.001 |
| TPINP (ng/mL) | | 78.17±4.37 | 67.15±6.76 | 5.415 | <0.001 |
| β-CTX(μg/mL) | | 0.92±0.16 | 0.74±0.13 | 3.936 | <0.001 |
| PTH (pg/mL) | | 83.46±10.63 | 73.75±8.25 | 3.288 | <0.001 |
| IGF-1(μg/L) | | 769.32±74.35 | 960.61±63.05 | 8.759 | <0.001 |

**Table 5 Variable assignment**

| Factors | Variable | Assignment |
| --- | --- | --- |
| X1 | Fracture history | 0=no, 1=yes |
| X2 | BGP | Measured value |
| X3 | TPINP | Measured value |
| X4 | β-CTX | Measured value |
| X5 | PTH | Measured value |
| X6 | IGF-1 | Measured value |

**Table 6 Factors affecting the prognosis of senile OS fracture**

| Factors | β value | Standard error | Wald χ2 value | P value | OR (95% CI) |
| --- | --- | --- | --- | --- | --- |
| X1 | 2.578 | 1.076 | 5.743 | 0.017 | 1.076(0.009～1.625) |
| X2 | 0.152 | 0.073 | 4.385 | 0.036 | 1.859(0.745～1.990) |
| X3 | 0.092 | 0.040 | 5.199 | 0.023 | 1.912(0.842～1.897) |
| X4 | 1.606 | 0.735 | 4.770 | 0.029 | 1.201(0.047～1.848) |
| X5 | 0.133 | 0.053 | 6.363 | 0.012 | 1.142(1.030～1.267) |
| X6 | -0.005 | 0.002 | 5.380 | 0.020 | 0.995(0.992～0.999) |


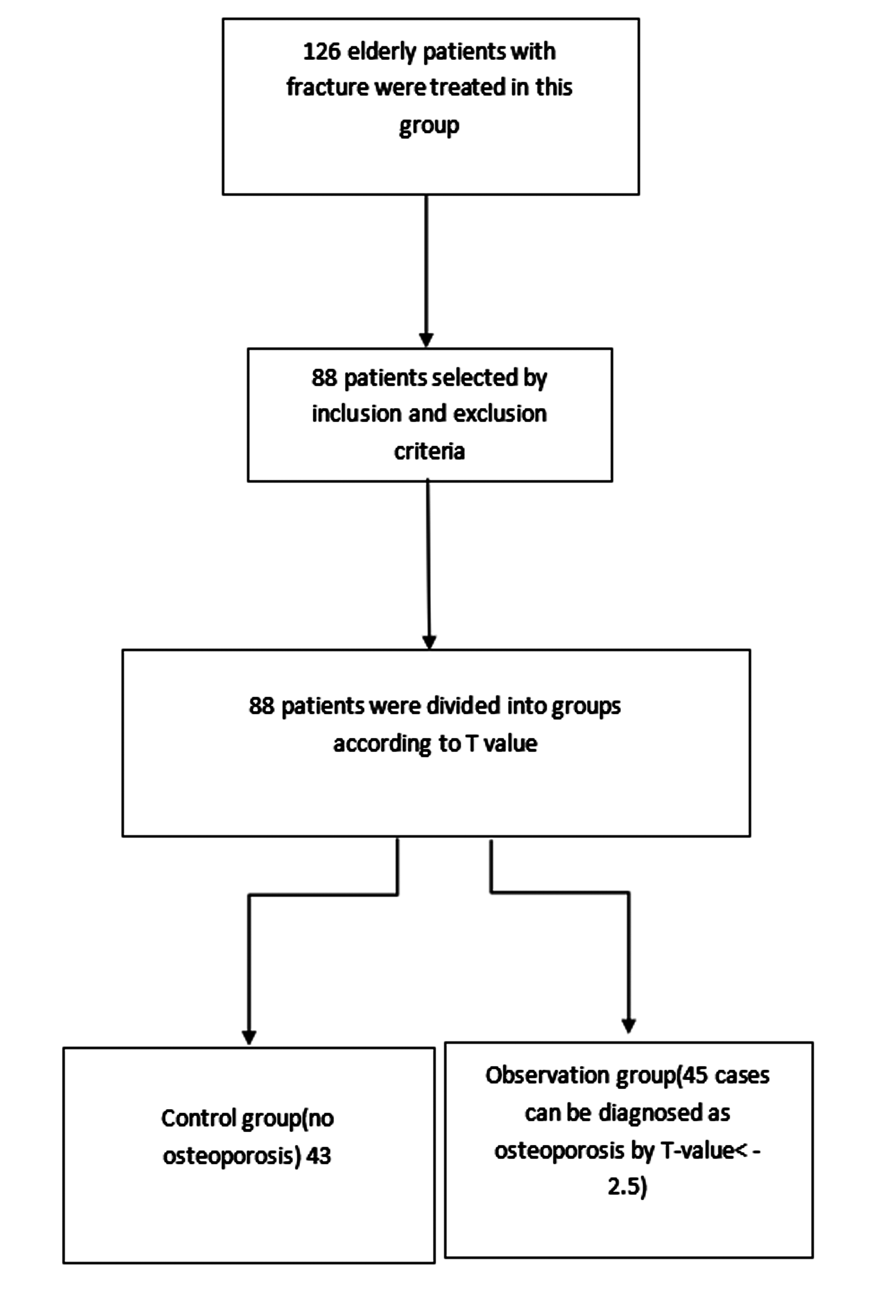


**Figure 1 The selection process of general data**

**
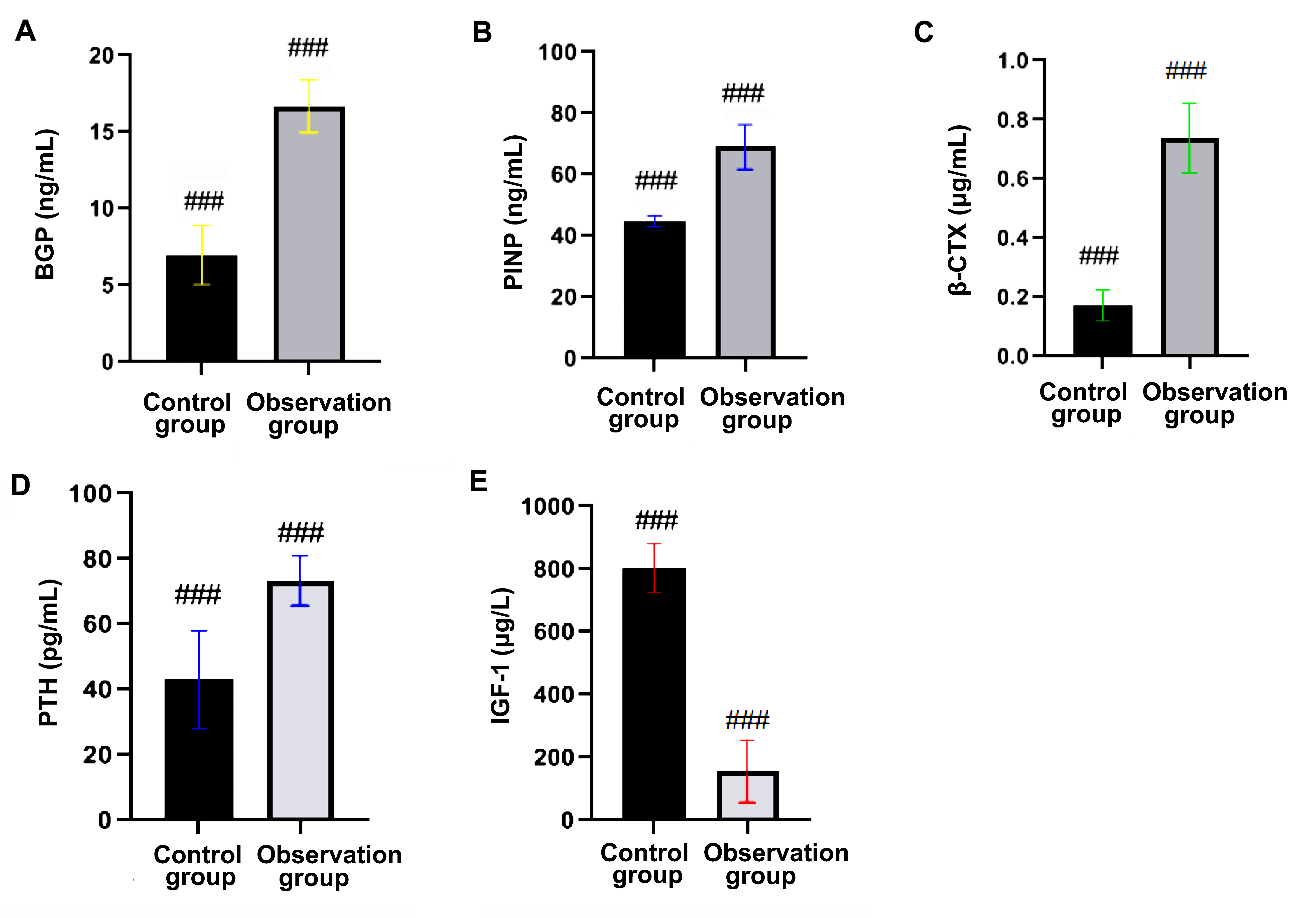
**

**Figure2 Comparison of biochemical indicators of bone metabolism levels**

A: Comparison of BGP level; B: Comparison of TPINP level; C: Comparison of β- CTX level;

D: Comparison of PTH level; E: Comparison of IGF-1 level

Note: ^*^ P<0.05 compared between two groups. BGP: boney containing protein; TPINP: total type 1 collagen amino acid extender peptide; β-CTX: β-Collagen special sequence; PTH: parathyroid hormone; IGF-1: insulin-like growth factor.


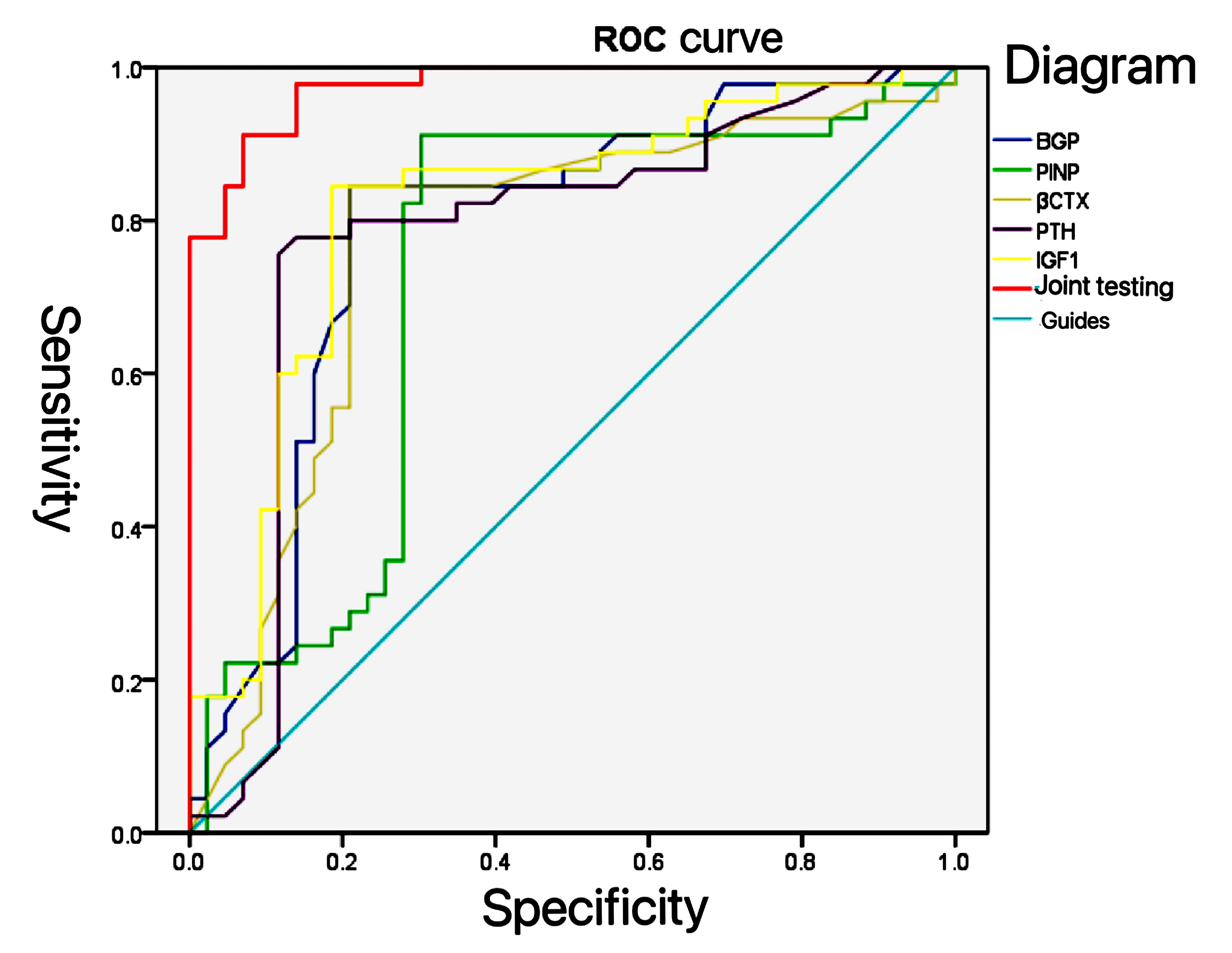


**Figure 3 ROC curve of bone metabolism biochemical indicators predicting the occurrence of OS fracture in the elderly**
